# Occupational Noise Annoyance Linked to Depressive Symptoms and Suicidal Ideation: A Result from Nationwide Survey of Korea

**DOI:** 10.1371/journal.pone.0105321

**Published:** 2014-08-21

**Authors:** Jin-Ha Yoon, Jong-Uk Won, Wanhyung Lee, Pil Kyun Jung, Jaehoon Roh

**Affiliations:** 1 The Institute for Occupational Health, Yonsei University College of Medicine, Seoul, Korea; 2 Department of Preventive Medicine and Public Health, Yonsei University College of Medicine, Seoul, Korea; 3 Graduate School of Public Health, Yonsei University College of Medicine, Seoul, Korea; University of Vienna, Austria

## Abstract

**Background:**

Noise, or undesirable sound, is one of the most common environmental stressors, and it can cause various health effects. Beyond the auditory consequences of occupational noise exposure, extra-auditory effects such as psychological problems have also been found. The aim of the current study is to elucidate the association between occupational noise annoyance and psychological symptoms, including symptoms of depression and suicidal ideation.

**Methods:**

A total of 10,020 participants (5,410 men and 4,610 women) were included in the current analysis, using data from the fourth Korean National Health and Nutrition Examination Survey (KNHANES). Self-report questionnaires were used to assess noise annoyance levels, depressive symptoms, and suicidal ideation. Odds ratios (ORs) and 95% confidence intervals (95% CIs) for psychosocial symptoms were calculated using multiple logistic regression models.

**Results:**

Compared to the no noise annoyance group, ORs (95% CI) of the severe annoyance groups were 1.58 (1.12–2.23) and 1.76 (1.29–2.40) in men and 1.49 (1.05–2.11) and 1.41 (1.01–1.97) in women for depressive symptoms and suicidal ideation, respectively. The ORs (95% CI) for severe noise annoyance in those with less than five hours of sleep were 2.95 (1.46–5.96) and 2.05 (1.01–4.16) in men and women, respectively, compared with those with no noise annoyance and a sleep time of more than five hours.

**Conclusion:**

Our study shows that occupational noise annoyance is significantly related to mental health, including depressive symptoms and suicidal ideation after controlling for individual and socio-demographic characteristics even with gender stratification. However, prospective studies with quantified noise exposure assessment were needed to elucidate the causality on the association between noise annoyance and psychological symptoms.

## Introduction

At its most basic, sound consists of physiological signals in the auditory system, enabling humans to communicate with both one another and the environment. However, external noise or undesirable sound is one of the most common environmental stressors, and it can result in various health consequences [1]. For example, noise-induced hearing impairment is a well-known occupational health hazard worldwide. Additionally, noise exposure is related to non-auditory effects [2], including annoyance, headache, sleep disturbance, and impaired cognitive development in children. Furthermore, acute noise exposure can cause vasoconstriction, and chronic noise exposure is related to hypertension and cardiovascular diseases [3].

The West London Survey [4] assessed 6,000 households on negative health effects related to living near a large London airport. The study revealed that high aircraft noise resulted in both acute and chronic irritability and depressive symptoms in local residents [4]. Following this discovery, additional research has suggested an association between noise exposure and mental health [5]. For example, a study from Japan identified a significant relationship between noise exposure and scores on a mental health assessment, including nervousness and depressive symptoms, with rates of mental illness increasing according to noise level [6]. This association remained significant even after adjusting for age, gender, marital status, housing type, and length of residence in the high exposure area [6]. However, another investigation that adjusted for socio-demographic variables did not find an association between aircraft noise and psychiatric hospital admission rates [7]. Additionally, the Caerphilly Study, based on prospective research, also found no association between mental disorder and traffic noise after controlling for socio-demographic factors [8]. These findings instead suggest that noisy settings reflect low socio-demographic environments, which themselves are linked to poor mental health. Thus, controversies exist as to whether noise exposure itself is related to mental illness after controlling for environmental and socio-demographic variables, such as income level and occupation. Furthermore, an epidemiology study on the topic did not include gender in a stratified analysis, despite the fact that there is a known association between gender and mental illness.

Generally, noise exposure in an occupational setting is more severe than in the general environment [9], and numerous reports exist on work-related hearing loss [10]. For example, almost one third of workers in Europe reported that, because the nose exposure was too loud, they would have to raise their voices to keep a conversation [9], [11]. 30–50% of workers in Asia are exposed to noise above 90 dB [12], which is loud enough to cause occupational stress. This type of occupational noise exposure has also been linked to a high risk of death from injury [13], suggesting that high noise exposure may reduce attention in the workplace, which can lead to injury. Additionally, severe stress is known to cause psychological problems, but studies on the effects of noise on psychological symptoms are often ignored in an occupational setting.

Our study aims to examine the association between noise exposure and psychological symptoms, including depressive symptoms and suicidal ideation, in an active working population. To clarify this association, data from a national representative survey, the Korea National Health and Nutrition Examination Survey (KNHANES), were assessed, adjusting for socio-demographic characteristics and stratified for gender.

## Methods

### Ethics statement

Participants provided written informed consent confirming their voluntary participation. All individual identifying records were anonymized prior to analysis. This survey was approved by the Institutional Review Board (IRB) of the Korea Centers for Disease Control and Prevention (KCDC) (IRB: 2007-02-CON-04-P; 2008-04EXP-01-C; 2009-01CON-03-2C).

### Fourth Korean National Health and Nutrition Examination Survey (KNHANES)

The KCDC conducted the fourth KNHANES from 2007 to 2009 [14], enrolling 13,800 households using stratified, multistage, probability sampling methods, based on 600 geographical population areas of Korea. Of the 10,067 economically active participants in the fourth KNHANES, 33 participants were excluded due to missing data on the noise exposure questionnaire, and 14 participants were excluded for missing data on depressive symptoms and suicidal ideation. Thus, data from 10,020 participants (5,410 in men, 4,610 in women) were used in the current analysis.

### Annoyance from occupational noise exposure (occupational noise annoyance) and occupation

Assessment of noise exposure in an occupational setting and personal perceptions of its effects were obtained from self-report questionnaires. Question for occupational noise exposure was “Are you exposed to noise loud enough that you would raise your voices to keep a conversation during work?” [9], [11], and who have answered yes to this question were asked to answer following question for personal perception of noise exposure. The answer to this question had three choices: No perception of occupational noise, perception of occupational noise without severe problems, and perception of occupational noise with severe problems. Hence, occupational noise annoyance was categorized as “none annoyance”, “mild annoyance” and “severe annoyance”, respectively.

Occupation type was categorized as white-, pink-, and blue-collar workers using a self-report questionnaire. White-collar workers included managers, senior officials, professionals, clerks, and skilled traders. Pink-collar workers were sales and customer service workers. Blue-collar workers included agriculture, fishery, forestry, crafts, and related trades, plant and machine operators, and elementary workers.

### Depressive symptoms and suicidal ideation

A separate set of questionnaires assessed workers for the presence of depressive symptoms and suicidal ideation during the past year. For depressive symptoms, we asked, “During the past year, have you felt feelings of sadness or hopelessness that persisted for at least two weeks and that disrupted your social life?” The questionnaire for suicidal ideation asked, “During the past year, have you ever felt that you were willing to die?” There were four possible options for each questionnaire (never, rarely, yes, and always), with yes and always being categorized as symptoms of depression and suicidal ideation. Psychological symptoms workers were defined as when a worker has at least one of the two psychological symptoms of depressive symptoms or suicidal ideation.

### Individual and household income and lifestyle factors

Income level was calculated using standardized methods of classifications by five-year age increments and gender compared with Korean standard income levels. Total family income was adjusted for the number of family members and was used to calculate quartile levels of household income. Hence, house hold income was categorized as low, middle-low, middle-high, and high income.

Smoking history was categorized as non-, former and current smokers. In the current study, “non-smokers” were defined as individuals who had smoked less than 100 cigarettes in their lifetime. Two or more occasions of drinking per week with seven or more glasses of alcohol in men, and two or more occasions drinking per week with five or more glasses of alcohol in women were defined as heavy alcohol drinking.

### Statistical analysis

Chi-squared tests and t-tests were used to compare group differences based on the presence of psychological symptoms. The odds ratios (ORs) and 95% confidence intervals (95% CIs) for psychological symptoms were estimated using a multivariate logistic regression model. Two-tailed p-values less than 0.05 were considered statistically significant.

## Results

### Socio-demographic characteristics according to psychological symptoms

Results for depressive symptoms and suicidal ideation are presented in Tables 1 and 2, respectively. The average age of suicidal ideation group in men, both the depressive symptom and the suicidal ideation group in women were significantly higher than that of non-symptomatic individuals. In regards to sleep, there were significantly higher rates of psychological symptoms in those who slept for five hours or less (depressive symptoms: men = 14.4%, women = 25.1%; suicidal ideation: men = 15.3%, women = 29.6%) than in those who got six or more hours of sleep (all p-values below 0.05). Low education was also associated with higher rates of psychological symptoms in both genders. For depression, those with education levels equal to or below primary school had rates of 13.2% and 86.8% for men and women, respectively, while those with university or above were 7.3% and 14.2%, respectively (all p-values under 0.05). Similar patterns emerged for education level and suicidal ideation. Blue-collar workers showed a higher proportion of depressive symptoms in women and higher suicidal ideation in both genders than the other types of workers (depressive symptoms: blue-collar women = 20.4%, white-collar women = 14.3%, p<0.01; suicidal ideation: blue-collar men = 11.5%, blue-collar women = 24.5%; white-collar men = 6.9%, white-collar women = 13.8%, all p-values under 0.05). Household income was also inversely related to both psychological conditions; the proportion of depressive symptoms in the low income group were 13.3% and 24.1% for men and women, respectively, while those in the high income group were 7.6% and 15.2%, respectively (all p values were below 0.05). Similarly, the proportion of suicidal ideation was higher in the low income group, with rates of 15.3% and 29.2% for men and women, respectively, compared with 7.2% and 13.3% in the high income group. In terms of lifestyle factors, female current smokers had higher rates of depressive symptom than non-smokers (23.2% vs. 17.7%, p = 0.023). The proportion of suicidal ideation in current smokers was also higher than in non-smokers in both sexes (current smoker vs. non-smoker: 10.8% vs. 9.1% in men, p = 0.048; 29.2% vs. 19.6% in women, p<0.01). Female heavy alcohol drinkers also had significantly higher rates of suicidal ideation compared with non-heavy drinkers (26.5% vs. 20.0%, p = 0.011).

**Table 1:** Basic characteristics of study population according to depressive symptom.

	Men			Women		
	Depressive symptom			Depressive symptom		
	Yes 512 (9.5%)	No 4898 (90.5%)	P	Yes 837 (18.2)	No 3773 (81.8)	P
*Age*	47.1±14.2	46.0±14.0	0.081	48.0±15.0	45.7±14.7	<.001
*Body mass index*	23.8±3.2	24.1±3.1	0.065	23.3±3.5	23.2±3.4	0.626
*Sleep duration*						
≤5hours	94 (14.4)	560 (85.6)	<.001	176 (25.1)	526 (74.9)	<.001
6 hours	134 (8.4)	1454 (91.6)		219 (18.7)	951 (81.3)	
7 hours	145 (9.0)	1465 (91.0)		219 (15.8)	1169 (84.2)	
≥8 hours	139 (8.9)	1419 (91.1)		223 (16.5)	1127 (83.5)	
*Education*						
Primary school	117 (13.2)	767 (86.8)	<.001	313 (22.3)	1094 (77.7)	<.001
Middle school	70 (10.3)	608 (89.7)		114 (19.8)	463 (80.2)	
High school	190 (9.5)	1810 (90.5)		250 (16.7)	1248 (83.3)	
Above university	135 (7.3)	1715 (92.7)		160 (14.2)	968 (85.8)	
*Occupation*						
White collar	142 (8.3)	1575 (91.7)	0.0713	198 (14.3)	1183 (85.7)	<.001
Pink collar	80 (9.3)	782 (90.7)		241 (18.7)	1046 (81.3)	
Blue collar	288 (10.3)	2501 (89.7)		394 (20.4)	1540 (79.6)	
*House hold income*						
Low	90 (13.3)	585 (86.7)	<.001	205 (24.1)	647 (75.9)	<.001
Middle low	141 (11.0)	1143 (89.0)		207 (17.3)	990 (82.7)	
Middle high	140 (8.7)	1479 (91.3)		221 (18.1)	1001 (81.9)	
High	132 (7.6)	1611 (92.4)		193 (15.2)	1075 (84.8)	
*Life style*						
Smoking habit						
none	108 (9.5)	1026 (90.5)	0.138	734 (17.7)	3425 (82.43)	0.023
former	144 (8.4)	1579 (91.6)		26 (21.9)	93 (78.2)	
current	260 (10.2)	2297 (89.8)		77 (23.2)	255 (76.8)	
Alcohol drinking						
Sever drinking	132 (10.2)	1159 (89.8)	0.280	783 (18.0)	3563 (82.0)	0.318
Others	380 (9.2)	3743 (90.8)		54 (20.5)	210 (79.5)	
*Noise annoyance*						
None	287 (8.5)	3080 (91.5)	0.001	571 (17.0)	2785 (83.0)	0.002
Mild	166 (10.3)	1452 (98.7)		215 (20.7)	826 (79.4)	
Severe	59 (13.8)	370 (86.3)		51 (23.9)	162 (76.1)	

**Table 2:** Basic characteristics of study population according to suicidal ideation.

	Men			Women		
	Suicidal ideation			Suicidal ideation		
	Yes 530 (9.8%)	No 4877 (90.2%)	P	Yes 941 (20.4%)	No 3669 (79.6%)	P
*Age*	48.9±15.3	45.8±13.9	<.0001	48.9±16.0	45.4±14.3	<.0001
*Body mass index*	23.7±3.2	24.1±3.1	0.0013	23.3±3.6	23.2±3.3	0.2269
*Sleep duration*				6.8±1.3	6.6±1.5	0.001
≤5hours	100 (15.3)	554 (84.7)	<.0001	208 (29.6)	494 (70.4)	<.0001
6 hours	149 (9.4)	1438 (90.6)		231 (19.7)	939 (80.3)	
7 hours	130 (8.1)	1483 (91.9)		235 (16.9)	1153 (83.1)	
≥8 hours	151 (9.7)	1402 (90.3)		267 (19.8)	1083 (80.2)	
*Education*						
Primary school	152 (17.2)	732 (82.8)	<.0001	383 (27.2)	1024 (72.8)	<.0001
Middle school	68 (10.0)	610 (90.0)		115 (20.0)	462 (80.1)	
High school	189 (9.5)	1811 (90.6)		280 (18.7)	1218 (81.3)	
Above university	121 (6.5)	1729 (93.5)		163 (14.4)	965 (85.6)	
*Occupation*						
White collar	119 (6.9)	1598 (93.1)	<.0001	190 (13.8)	1191 (86.2)	<.0001
Pink collar	87 (10.1)	775 (89.9)		273 (21.2)	1014 (78.8)	
Blue collar	321 (11.5)	2468 (88.5)		474 (24.5)	1460 (75.5)	
*House hold income*						
Low	103 (15.3)	572 (84.7)	0.0001	249 (29.2)	603 (70.8)	0.0001
Middle low	157 (12.2)	1127 (87.8)		280 (23.4)	917 (76.6)	
Middle high	133 (8.2)	1486 (91.8)		230 (18.8)	992 (81.2)	
High	126 (7.2)	1617 (92.8)		169 (13.3)	1099 (86.7)	
*Life style*						
Smoking habit						
none	103 (9.1)	1031 (90.9)	0.0477	814 (19.6)	3345 (80.4)	<.0001
former	150 (8.7)	1573 (91.3)		30 (25.2)	89 (74.8)	
current	277 (10.8)	2280 (89.2)		97 (29.2)	235 (70.8)	
Alcohol drinking						
Sever drinking	143 (11.1)	1148 (88.9)	0.0745	70 (26.5)	194 (73.5)	0.0113
Others	387 (9.4)	3736 (90.6)		871 (20.0)	3475 (80.0)	
*Noise annoyance*						
None	307 (9.1)	3060 (90.9)	0.0012	648 (19.3)	2708 (80.7)	0.0082
Mild	160 (9.9)	1458 (90.1)		240 (23.0)	801 (77.0)	
Severe	63 (14.7)	366 (85.3)		53 (24.9)	160 (75.1)	

In terms of noise level perceptions, rates of depressive symptoms were 8.5% and 17.0% for those who reported no annoyance, 10.3% and 20.7 at mild annoyance, and 13.8% and 23.9% with severe annoyance for men and women, respectively (p = 0.001 in men, 0.002 in women). Similarly, the proportion of suicidal ideation increased according to the severity of noise annoyance (none, mild, and severe annoyance: 9.1%, 9.9%, and 14.7% in men, p = 0.001; 19.3%, 23.0%, and 24.9% in women, p = 0.008).

### Odds ratios for depressive symptoms or suicidal ideation by noise annoyance (Table 3, 4)

Compared with the no noise annoyance group, ORs (95% CI) of the severe annoyance groups for depressive symptoms and suicidal ideation were 1.58 (1.12–2.23) and 1.76 (1.29–2.40) in men and 1.49 (1.05–2.11) and 1.41 (1.01–1.97) in women (model III), respectively, after adjusting for age, BMI, sleep time, education, occupation, household income, smoking habits, and alcohol.

**Table 3:** Odds ratio of depressive symptoms in a multivariate logistic regression models.

		Odds ratio (95% confidence interval)					
		Men			Women		
		Model I	Model II	Model III	Model I	Model II	Model III
Noise annoyance	none	1-	1-	1-	1-	1-	1-
	mild	1.24 (1.01–1.52)	1.25 (1.01–1.54)	1.21 (0.97–1.51)	1.29 (1.08–1.55)	1.29 (1.08–1.54)	1.26 (1.04–1.52)
	severe	1.79 (1.32–2.43)	1.81 (1.32–2.48)	1.58 (1.12–2.23)	1.50 (1.07–2.10)	1.49 (1.06–2.09)	1.49 (1.05–2.11)
Seeping time	≥8 hours	1-	1-	1-	1-	1-	1-
	7 hours	1.03 (0.80–1.32)	1.08 (0.84–1.39)	1.12 (0.86–1.45)	0.93 (0.76–1.14)	0.95 (0.78–1.17)	1.00 (0.80–1.24)
	6 hours	0.97 (0.75–1.24)	1.03 (0.80–1.33)	1.09 (0.83–1.42)	1.11 (0.91–1.37)	1.15 (0.93–1.41)	1.17 (0.94–1.46)
	≤5 hours	1.77 (1.32–2.33)	1.75 (1.31–2.32)	1.73 (1.27–2.34)	1.52 (1.21–1.92)	1.53 (1.21–1.92)	1.55 (1.22–1.98)
Education	primary school		1.84 (1.27–2.67)	1.82 (1.23–2.69)		1.57 (1.10–2.24)	1.58 (1.08–2.32)
	middle school		1.39 (0.96–2.01)	1.35 (0.91–2.01)		1.30 (0.92–1.82)	1.31 (0.91–1.89)
	high school		1.27 (0.98–1.65)	1.29 (0.98–1.69)		1.13 (0.88–1.46)	1.16 (0.89–1.51)
	above university		1-	1-		1-	1-
Occupation	white collar		1-	1-		1-	1-
	pink collar		0.89 (0.65–1.21)	0.87 (0.63–1.21)		1.16 (0.90–1.49)	1.05 (0.81–1.37)
	blue collar		0.80 (0.61–1.06)	0.82 (0.61–1.08)		1.03 (0.77–1.37)	1.02 (0.76–1.37)
House hold income	low		1.59 (1.15–2.20)	1.62 (1.15–2.27)		1.43 (1.11–1.84)	1.44 (1.10–1.88)
	middle low		1.36 (1.05–1.78)	1.34 (1.01–1.77)		0.98 (0.78–1.23)	1.02 (0.80–1.29)
	middle high		1.06 (0.82–1.37)	1.11 (0.85–1.44)		1.13 (0.91–1.40)	1.17 (0.93–1.46)
	high		1-	1-		1-	1-
Smoking	never			1-			1-
	former			0.79 (0.59–1.04)			1.24 (0.76–2.01)
	current			0.99 (0.77–1.28)			1.39 (1.01–1.92)
Alcohol drinking	others			1-			1-
	severe			1.05 (0.83–1.33)			0.95 (0.66–1.36)

All models adjusted for age and body mass index.

**Table 4:** Odds ratio of suicidal ideation in a multivariate logistic regression models.

		Odds ratio (95% confidence interval)					
		Men			Women		
		Model I	Model II	Model III	Model I	Model II	Model III
Noise annoyance	none	1-	1-	1-	1-	1-	1-
	mild	1.10 (0.90–1.35)	1.07 (0.86–1.32)	1.06 (0.85–1.31)	1.30 (1.09–1.54)	1.29 (1.08–1.53)	1.28 (1.07–1.52)
	severe	1.81 (1.34–2.44)	1.76 (1.29–2.39)	1.76 (1.29–2.40)	1.37 (1.01–1.91)	1.40 (1.01–1.95)	1.41 (1.01–1.97)
Seeping time	≥8 hours	1-	1-	1-	1-	1-	1-
	7 hours	0.83 (0.65–1.07)	0.89 (0.69–1.15)	0.88 (0.68–1.14)	0.82 (0.68–1.01)	0.85 (0.70–1.04)	0.87 (0.70–1.06)
	6 hours	1.03 (0.80–1.31)	1.14 (0.89–1.46)	1.13 (0.88–1.44)	1.00 (0.81–1.21)	1.03 (0.84–1.26)	1.05 (0.85–1.29)
	≤5 hours	1.66 (1.25–2.18)	1.68 (1.26–2.39)	1.65 (1.24–2.18)	1.52 (1.23–1.89)	1.50 (1.21–1.87)	1.42 (1.12–1.79)
Education	primary school		2.14 (1.49–3.08)	2.07 (1.44–2.98)		1.32 (0.94–1.86)	1.25 (0.86–1.80)
	middle school		1.23 (0.85–1.79)	1.19 (0.82–1.73)		0.98 (0.70–1.37)	0.91 (0.65–1.28)
	high school		1.30 (0.99–1.70)	1.28 (0.98–1.67)		1.05 (0.82–1.35)	1.00 (0.77–1.30)
	above university		1-	1-		1-	1-
Occupation	white collar		1-	1-		1-	1-
	pink collar		1.23 (0.90–1.68)	1.21 (0.88–1.65)		1.38 (1.08–1.77)	1.32 (1.03–1.69)
	blue collar		1.01 (0.76–1.34)	1.00 (0.75–1.32)		1.28 (0.97–1.69)	1.27 (0.96–1.67)
House hold income	low		1.58 (1.15–2.17)	1.57 (1.14–2.15)		2.07 (1.61–2.65)	2.01 (1.57–2.58)
	middle low		1.45 (1.11–1.89)	1.46 (1.12–1.90)		1.65 (1.32–2.07)	1.65 (1.31–2.06)
	middle high		1.04 (1.11–1.89)	1.04 (0.80–1.35)		1.40 (1.12–1.74)	1.39 (1.11–1.74)
	high		1-	1-		1-	1-
Smoking	never			1-			1-
	former			0.84 (0.64–1.11)			1.35 (0.87–2.10)
	current			1.12 (0.87–1.44)			1.66 (1.27–2.18)
Alcohol drinking	others			1-			1-
	severe			1.28 (1.03–1.58)			1.30 (0.96–1.78)

All models adjusted for age and body mass index.

In that model, all ORs (95% CI) for depressive symptoms and suicidal ideation in sleep duration less than or equal to five hours were 1.77 (1.32–2.33) and 1.76 (1.29–2.40) in men and 1.52 (1.21–1.92) and 1.41 (1.01–1.97) in women, respectively.

### Psychological symptoms (depressive symptoms or suicidal ideation) by occupational noise annoyance and sleep duration

**Figure 1:**
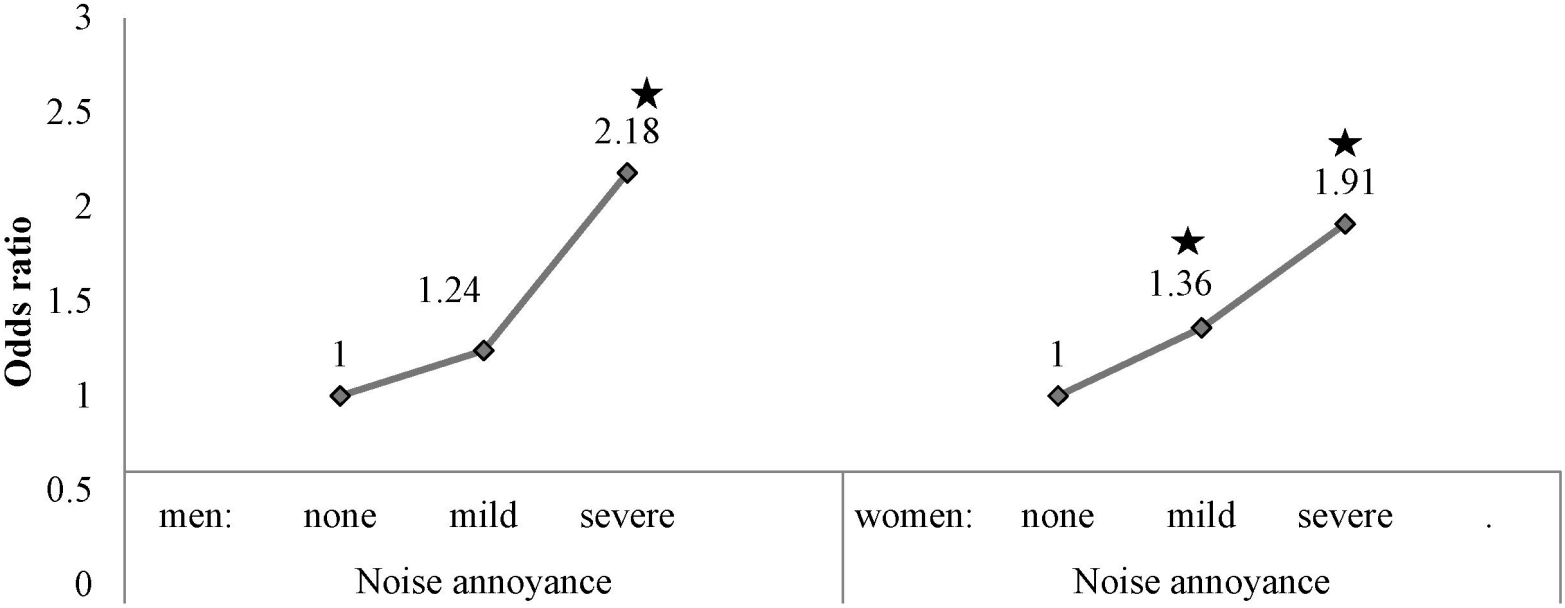
Odds ratio of suicidal ideation with depressed mood according to occupational noise annoyance (*p<0.05).

In multivariate regression analyses controlling for age, BMI, sleep duration, education, occupation, household income, smoking habits, and alcohol use, the ORs (95% CIs) for psychological symptoms (depressive symptoms or suicidal ideation) at severe annoyance were 2.18 (1.40–3.41) in men and 1.91 (1.27–2.86) in women (Figure 1).

**Figure 2:**
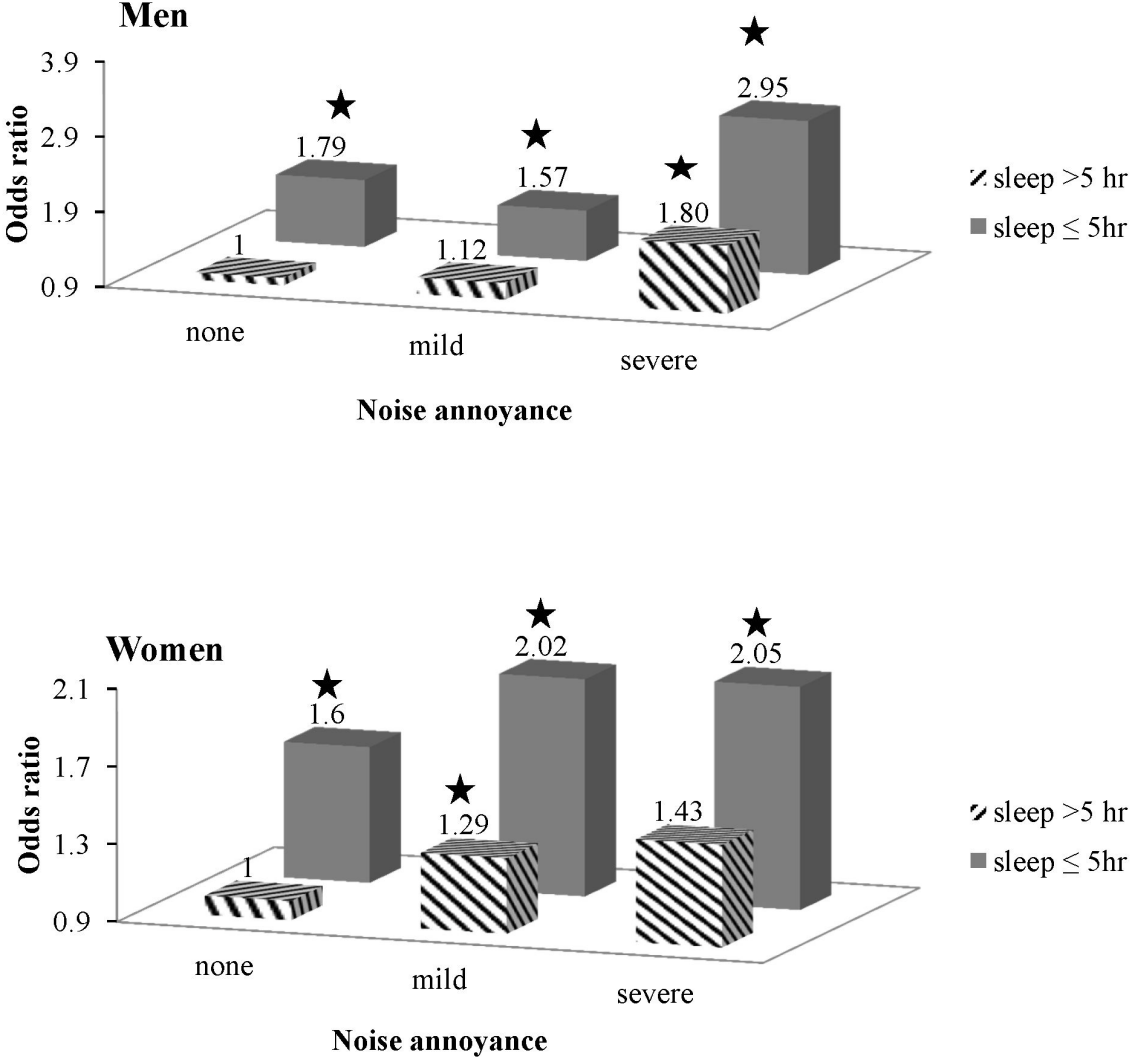
Risk of psychological symptoms (at least one of depressive symptoms or suicidal ideation) according to occupational noise annoyance and sleep duration (*p<0.05).

The interaction between sleep time and noise exposure was also calculated. In those with greater than five hours of sleep, the ORs (95% CI) of the severe noise annoyance compared with the no noise annoyance group were 1.80 (1.28–2.51) in men and 1.43 (0.98–2.07) in women. In both genders, the greatest ORs were observed in the severe noise annoyance group with less than five hours of sleep, with ORs (95% CI) of 2.95 (1.46–5.96) and 2.05 (1.01–4.16) times higher compared with the no noise annoyance with greater than five hours of sleep (Figure 2). There was no interaction effect of noise annoyance and sleep duration on psychological symptoms in the current study (p = 0.973 in men, 0.372 in women).

## Discussion

Our large, cross-sectional, nationwide study reported an important link between occupational noise annoyance and psychological symptoms, including depression and suicidal ideation. Those associations were not attenuated by adjusting for individual characteristics such as age, BMI, smoking habits, and alcohol drinking, as well as socio-demographic characteristics including education, occupation, and household income, even with gender stratification.

The current study has several limitations. First, because of the nature of cross-sectional studies, the direction of causality of occupational noise annoyance on psychological symptoms cannot be determined. Furthermore, depressive symptoms affect the individual appraisal of the noise exposure situation, and worker who have psychological problem could experience greater annoyance from noise exposure compare to healthy workers. This current relationship could be might due to this difference in perception. Although the results of our study are supported by the potential biological explanation of the impact of noise on the arousal system, prospective studies are needed to elucidate the causal relationship. Additionally, there was no information regarding actual sound level in terms of noise frequency and decibel level. However, as discussed above, the annoyance related to occupational noise could still serve as a simple but important measure when screening for health consequences from noise exposure.

Noise can be defined as undesirable sound [1]. Both the absolute level of sound and personal perception of noise levels are important factors that can affect human health [15]. As such, noise annoyance scales (ranging from “not at all” to “extremely”) are recommended by the International Commission on the Biological Effects of Noise [16]. In the current study, an assessment of noise exposure and its subjective effects (none annoyance, mild annoyance, and severe annoyance) were assigned by researchers using workers’ self-report questionnaire. Although we have no information on absolute noise exposure, the subjective level of noise annoyance captured by the current questionnaire is an important means for assessing the consequence of occupational noise on mental health, particularly as this measure was significantly related to psychological symptoms including depressive symptoms and suicidal ideation.

Some studies have suggested that sustained central autonomic arousal due to chronic noise exposure might be an important risk factor for psychological disorder [17]. For instance, dopamine, an essential neurotransmitter implicated in arousal and attention [18], has been shown to be disrupted upon exposure to noise [19]. Furthermore, dopamine has been linked to the pathophysiology of depression [20]; hence, there is a biological possibility that chronic noise exposure results in psychological abnormalities by disrupting the normal processes of arousal and the dopamine pathway. Hence, our current results are potentially supported by this biological mechanism.

Depressive symptoms and suicidal ideation are important risk factors for suicide attempts. For example, depressive symptoms persisting for more than two weeks is an essential component in the diagnosis of major depressive disorder according to both the Diagnostic and Statistical Manual of Mental Disorders (DSM-5) and the International Statistical Classification of Diseases and Related Health Problems (ICD-10) [21]. Additionally, attempted suicide is a key aspect of clinical emergency psychiatry [22]. Furthermore, the presence of suicidal ideation sharply increases the risk of a suicide attempt compared with non-suicidal ideation situations [23]. Hence, although the two questions in the current study did not cover all psychiatric diagnoses, our results suggest that these simple measures may be important psychological screening tools related to the health consequences of noise exposure.

A potential biological link has been suggested between noise exposure and poor quality of sleep [10]. In the current study, we were unable to assess sleep quality because lack of information; however, we did include sleep duration as a risk factor for psychological symptoms, with a sleep time of less than or equal to five hours related to psychological symptoms. However, there was no interaction between the effect of occupational noise annoyance and sleep duration on psychological symptoms. Hence, regardless of sleep time, our results suggest that noise exposure is an important and independent risk factor for psychological symptoms.

Previous studies have also reported that noise exposure and noise annoyance are linked to psychological symptoms [5], [6]. However, other investigations have shown that the association between noise exposure and psychological symptoms are not independent of socio-demographic factors [7], [8]. This might be due to the complex association between psychological abnormalities and socio-demographic characteristics [24]. Conversely, this lack of evidence might be related to the small sample size of the previous studies. However, our large cross sectional study showed that these associations were not affected after adjusting for individual and socio-demographic characteristics even with gender stratification. Moreover, these associations remained significant after stratification by sleep duration.

In general, there are gender differences associated with psychological symptoms and risk factors. For example, income level has a significant inverse relationship to suicidality in women but not in men [25], [26]. However, there were no gender differences in effect of income on psychological symptoms in the current study, and overall, there were no significant gender difference between occupational noise annoyance and psychological symptoms. This may be due to our study design, which only included currently employed individuals, thus tapping into the “healthy worker effect”.

In conclusion, our large, cross-sectional, nationwide study showed that occupational noise annoyance significantly related to mental health, including depressive symptoms and suicidal ideation. This link remained significant even after controlling for individual and socio-demographic characteristics even with gender stratification. However, prospective studies with quantified noise exposure assessment are needed to overcome our limitation of cross sectional design, and to elucidate the causality on the association between noise annoyance and psychological symptoms. To prevent both auditory effects and more general health consequences, such as psychological symptoms, from noise exposure, regulation strategies for occupational noise exposure are needed.
